# Development of a loop-mediated isothermal amplification detection assay for *Dictyocaulus viviparus* (Bloch, 1782) lungworm: DviLAMP

**DOI:** 10.3389/fvets.2024.1454065

**Published:** 2024-10-04

**Authors:** Sirapat Nak-on, Paul Campbell, Maha Mansour Shalaby, Jennifer McIntyre, Alistair Antonopoulos, Thapana Chontananarth, Roz Laing

**Affiliations:** ^1^Applied Parasitology Research Laboratory, Department of Biology, Faculty of Science, Srinakharinwirot University, Bangkok, Thailand; ^2^School of Biodiversity, One Health, and Veterinary Medicine, University of Glasgow, Glasgow, United Kingdom; ^3^James Watt School of Engineering, University of Glasgow, Glasgow, United Kingdom; ^4^Food Control Department, Faculty of Veterinary Medicine, Kafrelsheikh University, Kafr-El-Sheikh, Egypt; ^5^Kreavet, Kruibeke, Belgium; ^6^Research and Innovation Unit for Diagnosis of Medical and Veterinary Important Parasites, Faculty of Science, Srinakharinwirot University, Bangkok, Thailand

**Keywords:** nematode, lungworm, diagnosis, loop-mediated isothermal amplification, colorimetry, lateral flow system

## Abstract

The bovine lungworm, *Dictyocaulus viviparus* (Bloch, 1782), is highly pathogenic and disease outbreaks can be difficult to predict and manage. Rapid and accurate diagnosis is vital, but without a sensitive diagnostic test this remains challenging in clinical practice. High performance molecular detection tools are therefore required to improve the diagnosis of this parasite and promote the implementation of strategic control measures. Loop-mediated isothermal amplification (LAMP), a rapid DNA assay, offers potential for field-based detection. Here we report a novel LAMP assay (DviLAMP), that was designed to target the *D. viviparus* internal transcribed spacer 2 (ITS2) ribosomal DNA region. Firstly, genomic DNA was extracted from a single *D. viviparus* L_1_ larva to amplify and clone the ITS2 into the recombinant plasmid (DviITS2). The DviLAMP successfully detected the target, with results shown by gel electrophoresis and real-time analysis, in addition to point-of-care amenable end-point detection: colorimetry and lateral flow dipstick (LFD). Analytical sensitivity can detect 0.5 ng DviITS2 following 45 min of incubation at 64°C, increasing to just 1 pg following 90 min of incubation. Using the same primers, other nematodes of cattle, *Ostertagia ostertagi* and *Cooperia oncophora*, were also detectable both by gel electrophoresis and real-time. However, when FITC and biotin tagged primers were incorporated to adapt the DviLAMP to LFD end-point detection, the LFD showed specific detection of *D. viviparus*. Further development of DviLAMP as a point-of-care test could significantly improve the sensitivity of lungworm diagnosis in the field.

## Graphical abstract

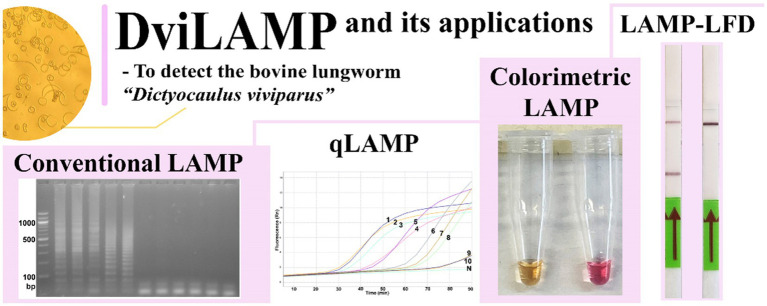

## Highlights

- A LAMP primer set was designed to detect *Dictyocaulus viviparus* ITS2 DNA.
- Conventional and colorimetric LAMP assays were tested for specificity and sensitivity.
- DviLAMP specifically detects *Dictyocaulus viviparus* on the lateral flow dipstick.

## 1 Introduction

*Dictyocaulus viviparus* (Bloch, 1782), the bovine lungworm, is the cause of parasitic bronchitis (dictyocaulosis) or “husk” and is a recognized cause of high morbidity and mortality in cattle. The disease occurs most frequently in calves in their first grazing season but can occasionally affect adult cattle. *Dictyocaulus viviparus* is found globally but is most prevalent in Europe, North and South America, and Australia (1). The economic cost of *D. viviparus* in the UK and Europe has recently been estimated at € 16 million and € 139 million, respectively (2).

Outbreaks of lungworm are sporadic and unpredictable but have been increasing in the UK (3). Due to the lack of a sensitive test, diagnosis relies on clinical signs, grazing history, and time of year. Coughing while at pasture is the major clinical sign but is not specific to lungworm (1). The classical diagnosis of *D. viviparus* infection is based on morphological identification of L_1_ larvae in the feces following Baermannisation (4, 5). However, false negatives are a problem with this approach due to the significant impact of storage temperature and time on larval recovery (6). Alternative molecular detection methods such as protein-based ELISA assays were developed to detect anti-lungworm antibodies (7) and antigen (8). The antibody level in bulk tank samples from dairy herds has been investigated as diagnostic strategy, but despite high specificity and a correlation between positive samples and disease outbreaks, this approach lacks sensitivity (4, 9). Techniques based on DNA detection by polymerase chain reaction (PCR) have also been developed to detect and identify *D. viviparus* from various ruminant hosts in a research setting (10).

The internal transcribed spacer 2 (ITS2) ribosomal DNA locus can be used for the identification of multiple strongyle species including *D. viviparus* by PCR (11–13). However, PCR-based approaches are time consuming and rely on specialized equipment, such as the thermocycler, that limits application in veterinary clinics or on farm. Loop-mediated isothermal amplification (LAMP) is an interesting technique that has been attracting growing attention for pathogen diagnostics (14–16). Several studies have been successful in developing LAMP assays for helminth parasites, with versatile and accessible options for read out such as colorimetry and lateral flow dipstick (LFD) (17–22).

Therefore, our study aimed to develop a new diagnostic tool for *D. viviparus* based on LAMP coupled with simple and point-of-care amenable end-point detection, including colorimetry and lateral flow. Real-time LAMP was used to confirm positive and negative results, in addition to determining the limit of detection in sensitivity analyses. Our proof-of-concept DviLAMP is the first step towards a sensitive and specific diagnostic tool for rapid and convenient detection of the bovine lungworm.

## 2 Materials and methods

### 2.1 DNA extraction

*Dictyocaulus viviparus* L_1_ larvae were recovered from cattle feces from a Scottish farm (23). Crude gDNA was extracted by lysis from individual larvae. Lysis buffer included 10 μL of Direct PCR Lysis Reagent (Cell, Viagen Biotech), 0.5 μL of 1 M DTT, and 0.1 μL of Proteinase K (100 mg/mL). The L_1_ was then incubated at 60°C for 120 min, followed by 85°C for 45 min to denature the Proteinase K. Individual *Ostertagia ostertagi* (Stiles, 1892) and *Cooperia oncophora* (Railliet, 1898) L_3_ from cattle feces from Scottish farms were processed in the same manner to obtain gDNA. Extracted DNA was diluted 20-fold and stored at −20°C.

### 2.2 Plasmid preparations

To amplify the ITS2 region, a PCR reaction (final volume 20 μL) was set up, including 4 μL of 5X Phusion Green GC Buffer, 0.4 μL of 10 mM dNTPs solution (N0447S, NEB), 0.4 μL of 10 μM of each generic forward and reverse primer [ITS2GF and ITS2GR, respectively, from a previous publication (24)] (Eurofins Genomics), 0.2 μL of 2 U/μL Phusion High-Fidelity DNA Polymerase (F-534S, Thermo Scientific), 1 μL of DNA template, and DEPC H_2_O (AM9906, Ambion, Invitrogen). A thermocycler (Biometra TAdvanced, Analytik Jena) with the following thermal conditions was used: denature at 98°C for 30 s, 40 cycles of 98°C for 20 s, annealing temperatures (61°C for first 15 cycles, and 58°C for later 25 cycles) for 20 s, and 72°C for 20 s, followed by a final extension at 72°C for 5 min. The PCR product was visualized by 2% agarose gel (NBS-AG500, NBS Biologicals) electrophoresis under UV (FireReader, UVITEC) to verify the PCR amplicon size (~593–596 bp for *D. viviparus*; ~378–381 bp for *O. ostertagi* and *C. oncophora*). Next, the remaining volume (15 μL) was purified using the Monarch PCR and DNA Cleanup Kit (T1030S, NEB). Purified PCR products from five individual L_1_ samples were pooled. For A tailing, 2 μL of the purified amplicon (~10 ng/μL) was mixed with 2 μL of 5X GoTaq Flexi Reaction buffer, 2 μL of GoTaq Flexi DNA Polymerase (Promega), 2 μL of 1 mM dATP (10216018, Invitrogen), 0.6 μL of 25 mM MgCl_2_, and 1.4 μL of DEPC H_2_O, and then incubated at 72°C for 20 min. The PCR product was ligated into the plasmid (pCR^™^ TOPO^™^ vector) and the recombinant plasmid was transformed into competent cells according to the TA Cloning Kit with One Shot^™^ TOP10 *E. coli* (Invitrogen) manufacturer’s instructions. After incubation on LB plates at 37°C overnight, the recombinant plasmid containing colonies were identified by blue-white colony selection, followed by PCR to check the plasmids contained the desired ITS2 product. The recombinant plasmid (DviITS2) was extracted using a Monarch Plasmid Miniprep Kit (T1010S, NEB) and the DNA concentration was evaluated by Qubit (high sensitivity dsDNA quantification assay).

### 2.3 DNA sequencing and species confirmation

Plasmids were sequenced by TubeSeq Supreme (Eurofins Genomics), using both forward and reverse primers. Sequences for each clone were aligned and assembled using MEGA 11 software and the species confirmed using BLASTn (National Center for Biotechnology Information (NCBI), https://blast.ncbi.nlm.nih.gov/Blast.cgi). The 5.8S and 28S rDNA regions were annotated based on a previous study (24) to identify the precise ITS2 region. Then, the assembled DNA sequences were aligned by MEGA 11 software, exported to FASTA format, and uploaded into MultAlin online software [(25), http://multalin.toulouse.inra.fr/multalin/multalin] to generate a plain text DNA sequence alignment. DNA sequences from this study were published under accession numbers: PP970511-PP970515 for *D. viviparus*; PP968975-PP968976 for *O. ostertagi*; and PP968977 for *C. oncophora*.

### 2.4 LAMP primer design

**Figure 1:**
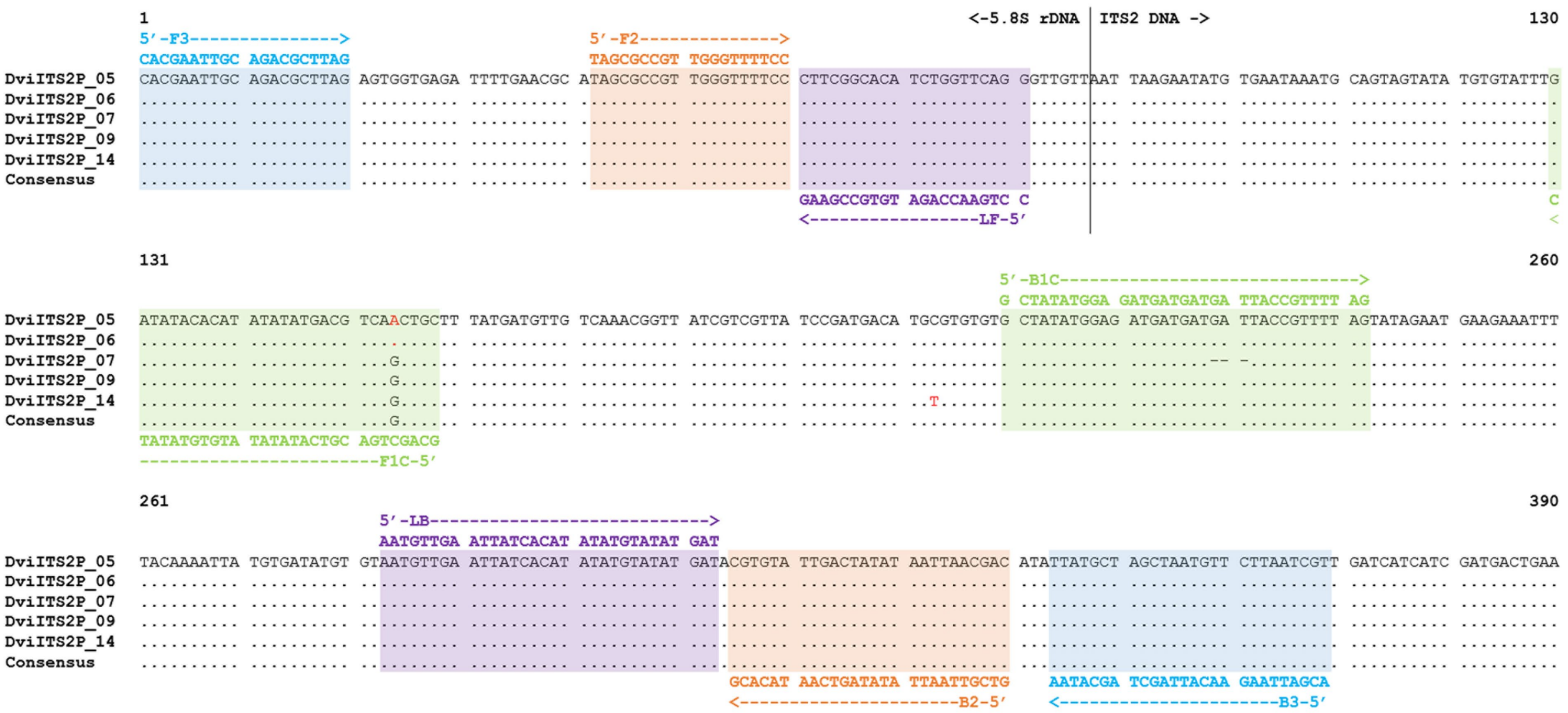
ITS2 with partial 5.8S rDNA sequence alignment of *Dictyocaulus viviparus* (clones 5, 6, 7, 9, and 14) highlighted with different DviLAMP primer DNA sequences; variable sites in the DNA sequence are presented as red letters.

A consensus sequence of the *D. viviparus* ITS2 region with partial 5.8S rDNA sequence was derived from the five picked clones (Figure 1). Primer design was carried out with Geneious Prime software (Version 2023.2.1, Dotmatics). The forward outer primer was designed manually, with a fixed start at the 5′ end of the 5.8S rDNA sequence, then imported into PrimerExplorer V5.^1^ The loop primers were also designed manually using Geneious Prime (Version 2023.2.1, Dotmatics). General properties of the primers were investigated *in-silico* using the OligoAnalyzer Tool [Integrated DNA Technologies (IDT), https://sg.idtdna.com/calc/analyzer] to assess the following criteria: length, Tm, ΔG, hairpin, homodimer, and heterodimer formation. The selected DviLAMP primers for this study (Table 1) were ordered from IDT and Eurofins Genomics in lyophilized form.

**Table 1:** DviLAMP primers for *Dictyocaulus viviparus* lungworm detection: [BIO], biotin; [FITC], fluorescein-5-isothiocyanate; and bp, base pair.

Primer name	Nucleotide sequence (5′–3′)	Length (bp)
DviLAMP-F3^a^	5′-CACGAATTGCAGACGCTTAG-3′	20
DviLAMP-B3^a^	5′-ACGATTAAGAACATTAGCTAGCATAA-3′	26
DviLAMP-FIP^a^ (F1C-F2)	5′-GCAGCTGACGTCATATATATGTGTATATC-TAGCGCCGTTGGGTTTTCC-3′	48
DviLAMP-BIP^a^ (B1C-B2)	5′-GCTATATGGAGATGATGATGATTACCGTTTTAG-GTCGTTAATTATATAGTCAATACACG-3′	59
DviLAMP-LF^a^	5′-CCTGAACCAGATGTGCCGAAG-3′	21
DviLAMP-LB^a^	5′-AATGTTGAATTATCACATATATGTATATGAT-3′	31
DviLAMP-FIPL^b^	5′-[BIO]-GCAGCTGACGTCATATATATGTGTATATC-TAGCGCCGTTGGGTTTTCC-3′	48
DviLAMP-BIPL^b^	5′-[FITC]-GCTATATGGAGATGATGATGATTACCGTTTTAG-GTCGTTAATTATATAGTCAATACACG-3′	59
DviLAMP-LFL^b^	5′-[FITC]-CCTGAACCAGATGTGCCGAAG-3′	21
DviLAMP-LBL^b^	5′-[BIO]-AATGTTGAATTATCACATATATGTATATGAT-3′	31

a Ordered from IDT.

b Ordered from Eurofins Genomics.

### 2.5 Conventional and colorimetric LAMP

The DviLAMP primer mix was prepared in a 10X mixture solution, including 16 μM each of forward inner primer (FIP) and backward inner primer (BIP), 8 μM each of forward loop primer (LF) and backward loop primer (LB), and 4 μM each of forward outer primer (F3) and backward outer primer (B3), prior to using in colorimetric and real-time LAMP. For colorimetric LAMP, a 1X LAMP-reaction mixture (12.5 μL final volume) containing 6.25 μL of WarmStart Colorimetric LAMP 2X Master Mix (M1800S, NEB), 2.5 μL of 5 M betaine (Sigma-Aldrich), 1.25 μL of 10X DviLAMP primer mix, 1.5 μL of DEPC H_2_O, and 1 μL of the DNA template (20 ng/μL–1 pg/μL DviITS2 plasmid) was used. For each batch of experiments, a negative control was run using 1 μL DEPC H_2_O in place of the DNA template. The LAMP assay was performed in a thermocycler (Biometra TAdvanced, Analytik Jena) at 64°C for three different reaction durations: 45, 60, and 90 min, with a final 80°C incubation for 10 min to terminate the reaction. The post-reaction tubes were left until they reached room temperature (~20–25°C), then were mixed well and spun down. The solution color (yellow as positive; or pink as negative) was assessed in good light on a white paper background and recorded by taking a photo with an iPad camera. After recording the color change, 5 μL of LAMP product was then visualized using 2% agarose gel electrophoresis.

#### 2.5.1 Real-time LAMP

For real time LAMP, the 12.5 μL reaction mixture (1X) contained 1.25 μL of 10X isothermal amplification buffer, 1.25 μL of 10X DviLAMP primer mix, 1.75 μL of 10 mM/base dNTPs, 0.75 μL of 100 mM MgSO_4_, 2.5 μL of 5 M Betaine, 0.5 μL of 8 U/μL *Bst* 2.0 WarmStart DNA Polymerase (M0538S, NEB), 0.25 μL of 50X LAMP Fluorescent Dye (B1700S, NEB), 0.375 μL of 1 μM passive reference dye (600530, Agilent), 2.875 μL of DEPC H_2_O, and 1 μL of the DNA template (20 ng/μL–1 pg/μL DviITS2 plasmid or H_2_O). The thermal profile was controlled under AriaMx Real-time PCR System (G8830A, Agilent Technologies), at 64°C for 60 min for specificity validation and 90 min for sensitivity determination, then 80°C for 10 min. High resolution melt (HRM) analysis of the LAMP product was then performed at 95°C for 30 s, 65°C for 30 s, and 95°C for 30 s, with 0.5°C resolution and 5 s for soak time. FAM and ROX channels were selected for fluorescence detection and as the reference dye, respectively. The real-time result with three technical replicates for each DNA template was recorded and analyzed using AriaMx software (Agilent). Normalized fluorescence with the reference dye (Rn) and the first derivative of the normalized fluorescence multiplied by −1 [−Rn´(T)] values were measured to investigate the fluorescence signal data (as *Y*-axis) of the amplification plot and melt curve, respectively.

#### 2.5.2 Lateral flow LAMP

Both conventional and colorimetric LAMP reactions gave the same result on lateral flow dipstick (LFD) so were used interchangeably in this study. For colorimetric LAMP, the reaction mixture for LFD was the same as section 2.5 except in this case, tagged primers were used, where biotin and fluorescein-5-isothiocyanate (FITC) tagged primers (FIPL, BIPL, LFL, and LBL) replaced untagged primers (FIP, BIP, LF, and LB, respectively), as presented in Table 1. For conventional LAMP with LFD (without colorimetry), the 12.5 μL final volume contained 1.25 μL of 10X isothermal amplification buffer, 1.25 μL of 10X DviLAMP primer mix (a pair of biotin and FITC tagged primers), 1.75 μL of 10 mM/base dNTPs (N0447S, NEB), 0.75 μL of 100 mM MgSO_4_, 2.5 μL of 5 M Betaine, 0.5 μL of 8 U/μL *Bst* 2.0 WarmStart DNA Polymerase (NEB), 3.5 μL of DEPC H_2_O, and 1 μL of the DNA template (20 ng/μL–10 pg/μL DviITS2 plasmid) or H_2_O (for negative control). For both LAMP assays the thermal profile was the same as described in section 2.5. After incubation, 5 μL of the LAMP product was mixed with 45 μL of a commercial lateral flow buffer (from the LFD kit, Milenia Genline HybriDetect, Germany) in a 1.5 mL microcentrifuge tube. The LFD was submerged in the mixture for 5 min and then removed to record the result. To select the optimum pair of biotin/FITC tagged primers, four schemes trialing different positions of biotin and FITC tagging, including (I) [BIO]-FIPL and [FITC]-BIPL; (II) [FITC]-LFL and [BIO]-LBL; (III) [BIO]-FIPL and [FITC]-LFL; and (IV) [BIO]-LBL and [FITC]-BIPL, with LAMP incubation at 64°C for 60 min, were tested with LFD (see results section 3.2).

### 2.6 Analytical sensitivity and specificity of the LAMP assay

To test the analytical sensitivity, DviLAMP with 45, 60, and 90 min of incubation at 64°C was applied to various DviITS2 plasmid DNA concentrations from 20 ng to 1 pg. To test the analytical specificity, *O. ostertagi* and *C. oncophora* ITS2 plasmids were incorporated separately, and together, with the DviITS2 plasmid and tested by the DviLAMP with 60 min of incubation at 64°C. The results were analyzed by colorimetry, gel electrophoresis, real-time, and LFD.

## 3 Results

### 3.1 DNA sequences

In this study, we made use of seven ITS2 sequences with flanking 5.8S and 28S rDNA regions from three species; five *D. viviparus* clones (593–596 bp with 97.22–100% identity to other *D. viviparus* DNA sequences in the NCBI database) and one sequence each for *O. ostertagi* (378 bp with 98.14–100% identity to other *O. ostertagi* DNA sequences in the NCBI database) and *C. oncophora* (381 bp with 99.21–99.74% identity to other *C. oncophora* DNA sequences in the NCBI database). Two DNA sequences from *D. viviparus* (clone 5 and 6) were 100% identical so only four variants were found. The predicted primer hybridization sites on the DNA alignments are shown in Figure 1 (*D. viviparus*) and Supplementary Figure S1 (*O. ostertagi* and *C. oncophora*). BLASTn results and DNA sequence annotations are shown in Supplementary Table S1.

### 3.2 Analytical sensitivity

**Figure 2:**
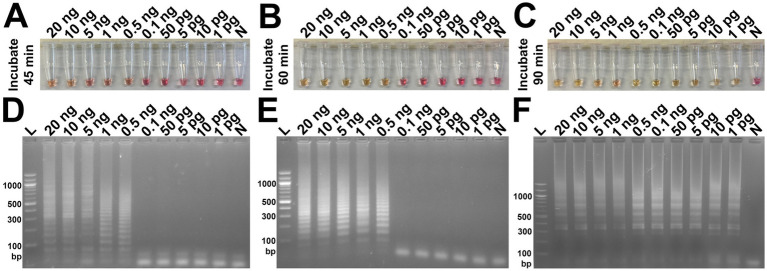
Analytical sensitivity test of the DviLAMP based on colorimetry with naked eye observation **(A–C)** and gel electrophoresis **(D–F)** with different incubation periods, including 45 min **(A,D)**, 60 min **(B,E)**, and 90 min **(C,F)**; and various DNA concentrations (20 ng to 1 pg): L, DNA ladder; N, negative control.

**Figure 3:**
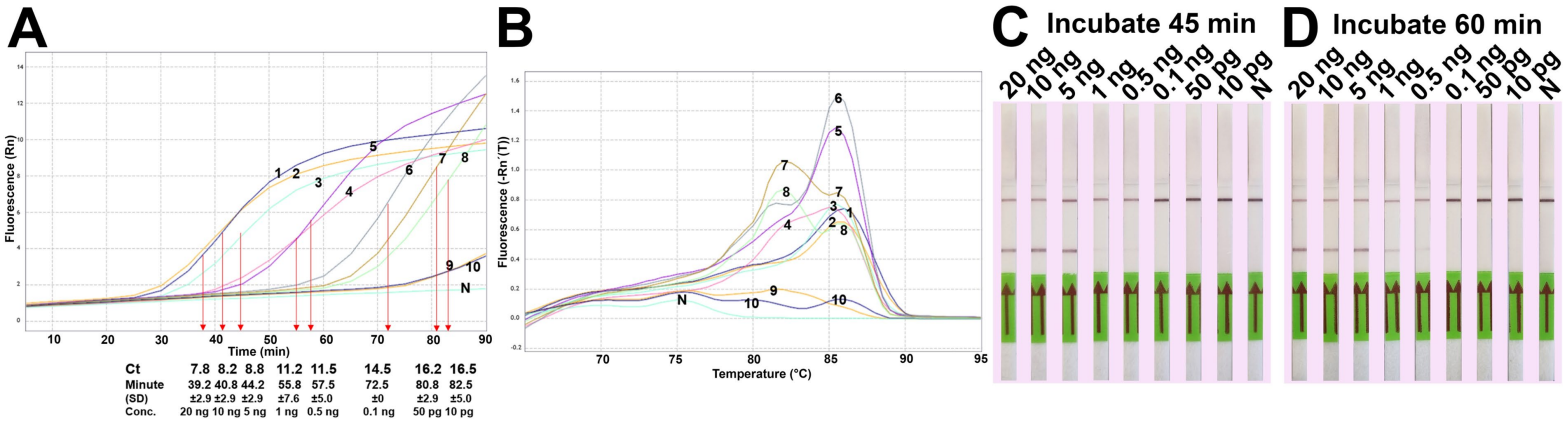
DviLAMP with real-time detection **(A)**, melting curve plot **(B)** and lateral flow **(C,D)**. The real-time fluorescence signal was calculated from three replicates. Ct values and time of the highest change in the fluorescence were calculated and indicated in the amplification plot. Numbering in the amplification plot indicates the DviITS2 plasmid DNA concentrations as follows: 1 = 20 ng; 2 = 10 ng; 3 = 5 ng; 4 = 1 ng; 5 = 0.5 ng; 6 = 0.1 ng; 7 = 50 pg; 8 = 10 pg; 9 = 5 pg; and 10 = 1 pg.

The analytical sensitivity results in this study are summarized in Table 2. After 45 min incubation, colorimetric DviLAMP detected down to 0.5 ng of DviITS2 plasmid, turning from pink to pale yellow/orange (Figure 2A). The color change to a brighter yellow continued until 60 min (Figure 2B). Extending incubation of the DviLAMP to 90 min increased sensitivity, allowing detection of 1 pg of DviLAMP plasmid, which was the lowest DNA concentration tested in this study (Figure 2C). All positive colorimetry results were supported by the appearance of a smear-like ladder on a gel, typical of a LAMP reaction, and there were no products observed for any negative (pink color mixture) result (Figure 2). The real-time amplification plot also supported the sensitivity test (Figure 3). Florescence signals for 20 ng to 0.5 ng began to increase in exponential phase from ~35–45 min, with the late linear or plateau phase at 60 min or more. Increasing florescence signal was detected for all DNA concentrations and the Ct value or the time of the highest change in signal (exponential phase) (Figure 3A), could be calculated for all concentrations except for 5 pg and 1 pg. However, the florescence signals for these two lowest DNA concentrations were detected by 90 min. HRM analysis (Figure 3B) confirmed the varied sizes of LAMP products from different DNA concentrations.

**Table 2:** Analytical sensitivity of the DviLAMP to detect DviITS2 in various DNA concentrations.

LAMP assay	DNA concentration									
	20 ng	10 ng	5 ng	1 ng	0.5 ng	0.1 ng	50 pg	10 pg	5 pg	1 pg
Conventional LAMP										
45 min	+	+	+	+	+	−	−	−	−	−
60 min	+	+	+	+	+	−	−	−	−	−
90 min	+	+	+	+	+	+	+	+	+	+
Colorimetry LAMP										
45 min	+	+	+	+	+	−	−	−	−	−
60 min	+	+	+	+	+	−	−	−	−	−
90 min	+	+	+	+	+	+	+	+	+	+
LAMP-LFD										
45 min	+	+	+	+	+	−	−	−		
60 min	+	+	+	+	+	−	−	−		

+ = positive result; − = negative result.

**Figure 4:**
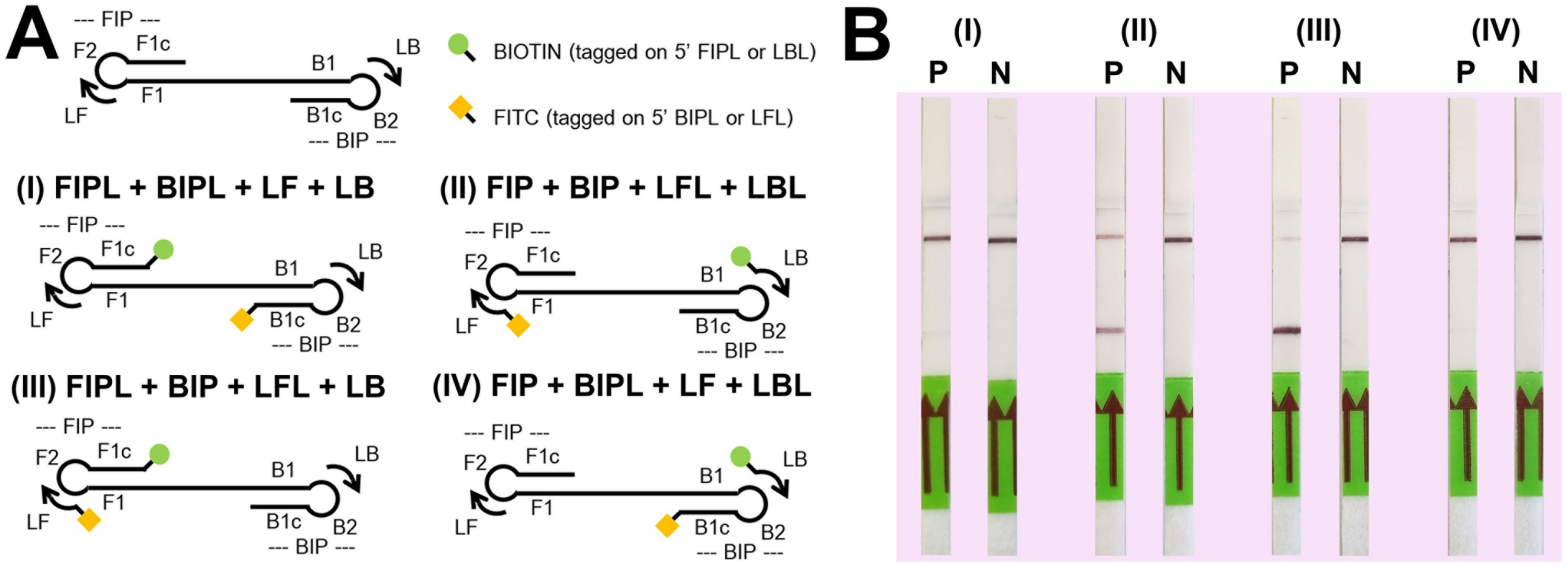
Biotin (BIO) and fluorescein-5-isothiocyanate (FITC) position tagging test. Graphics for four tagged position schemes **(A)** including (I) [BIO]-FIPL and [FITC]-BIPL; (II) [FITC]-LFL and [BIO]-LBL; (III) [BIO]-FIPL and [FITC]-LFL; and (IV) [BIO]-LBL and [FITC]-BIPL. The P (positive, 10 ng of *Dictyocaulus viviparus* as DNA template) and N (negative, H_2_O) results on LFD for each scheme after amplification for 60 min **(B)**.

For the LFD assay, we tested four pairs of biotin and FITC labelled primers (Figure 4A). Test line appearance could be observed clearly from two schemes (Figure 4B), which were [FITC]-LFL and [BIO]-LBL (scheme II); and [BIO]-FIPL and [FITC]-LFL (scheme III). For scheme III, the test line was stronger than scheme II but the control line was faint. Therefore, we decided to choose the biotin-FITC tagged primers from scheme II to test the sensitivity of DviLAMP-LFD for 45 min and 60 min reaction time (Figures 3C,D). The test line was visible from 20 ng to 0.5 ng of DNA for both incubation times, indicating the limit of detection for the DviLAMP was 0.5 ng. For the lowest amounts of input DNA, 1 ng and 0.5 ng, lines were visible but faint after 45 min of incubation but were clearly visible after 60 min of incubation. All four variants of the *D. viviparus* ITS2 DNA in this study were detected by DviLAMP (Supplementary Figure S2).

### 3.3 Analytical specificity

**Figure 5:**
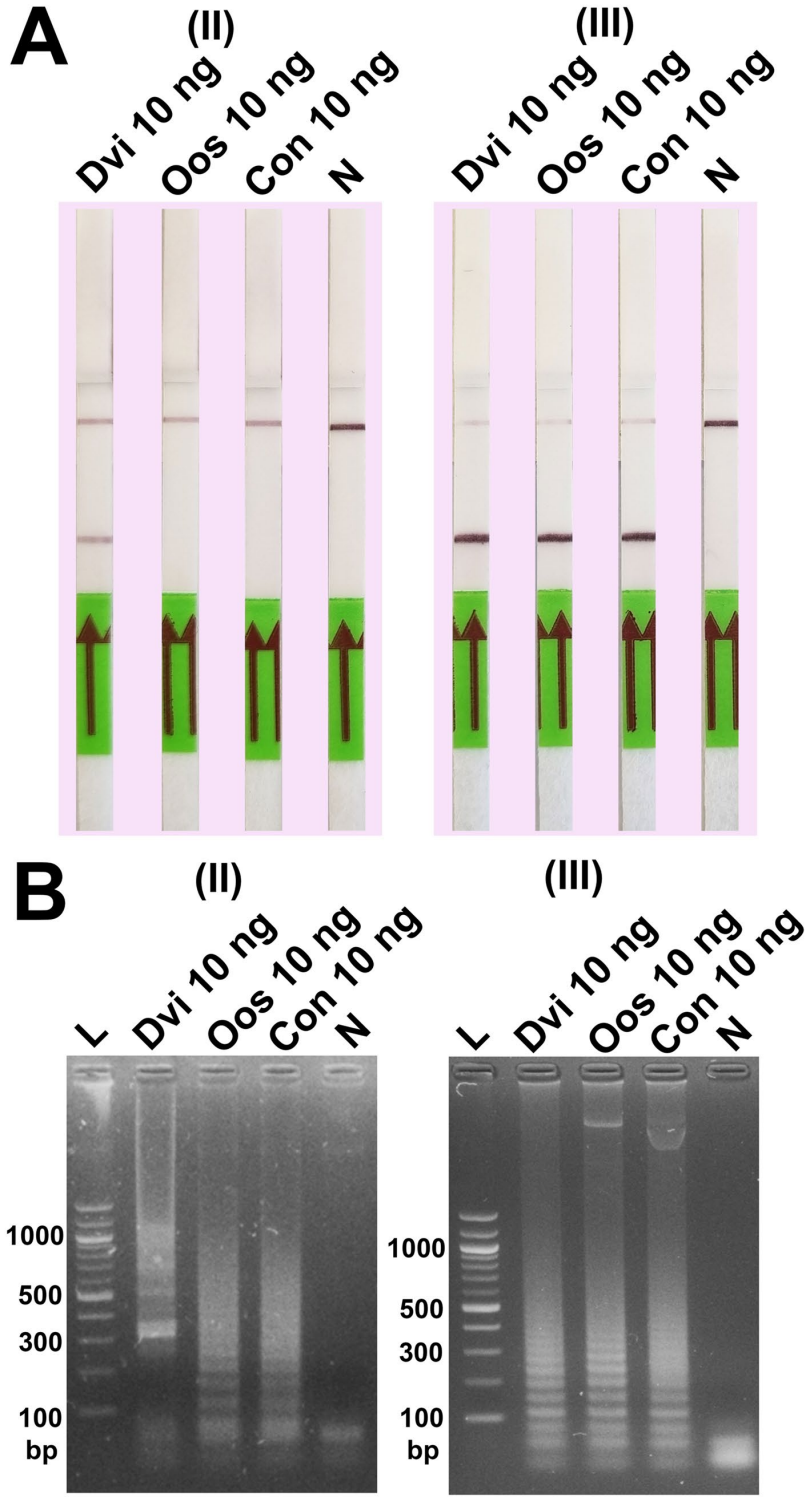
Specificity test of the DviLAMP with two biotin-FITC tagged position schemes, (II) [FITC]-LFL and [BIO]-LBL; and (III) [BIO]-FIPL and [FITC]-LFL, showing the results by LFD **(A)** and gel electrophoresis **(B)** for *Dictyocaulus viviparus* (Dvi), *Ostertagia ostertagi* (Oos), and *Cooperia oncophora* (Con) after amplification for 60 min.

Our results showed that changing the position of the biotin and FITC labels impacted the ability of DviLAMP with LFD to detect the target (*D. viviparus*) and non-target (*O. ostertagi* and *C. oncophora*) ITS2 plasmids (Figure 5). There was a notable difference in the specificity of the assay dependent on which primers were labelled. While all three parasites were detected by gel electrophoresis in both schemes II and III, and by LFD in scheme III, only *D. viviparus* was detected by the LFD in scheme II. These findings support the choice of biotin and FITC tagged primers in scheme II for specific detection of *D. viviparus*.

**Figure 6:**
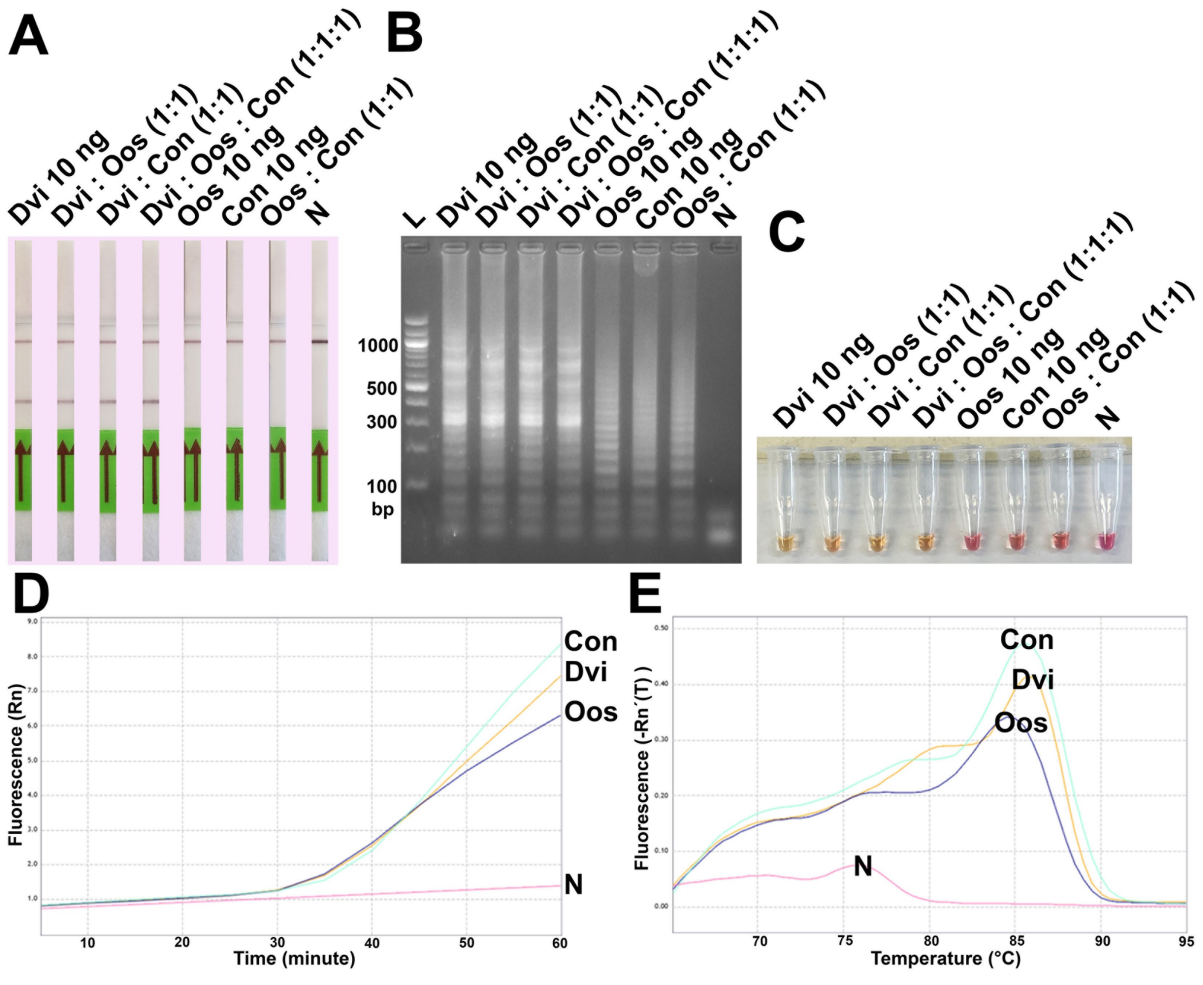
Analytical specificity test of the DviLAMP by LFD **(A)**, gel electrophoresis **(B)**, and colorimetry **(C)**; the amplification plot **(D)** and the melting plot **(E)** showing the fluorescence increasing for *Dictyocaulus viviparus* (Dvi), *Ostertagia ostertagi* (Oos), and *Cooperia oncophora* (Con) for 60 min of the incubation. The amplification plot and melting plot show the results for each species individually from three technical replicates.

Finally, ITS2 plasmids from all three species were combined and used as template for DviLAMP (Figure 6). Whether mixed in a 1:1 ratio with either *O. ostertagi* or *C. oncophora* plasmids, or in a 1:1:1 reaction mixture with both, *D. viviparus* DNA could still be specifically detected with LFD and colorimetry (LFD test line appearance and yellow color change, Figures 6A,C, respectively). While gel electrophoresis and real time LAMP amplification showed amplified LAMP product for all three species when tested individually (Figures 6B,D), this was not reflected in the LFD or colorimetry results. HRM peaks for these species presented no obvious difference (Figure 6E). No LFD test line was visible for *O. ostertagi* or *C. oncophora* and colorimetry showed no change for *O. ostertagi* and a slight change for *C. oncophora* to an orange shade of pink (Figure 6C). Therefore, only *D. viviparus* can be detected and differentiated from a negative result by colorimetry and LFD, even in the presence of DNA of other species.

## 4 Discussion

A commercial vaccine using radiation-attenuated *D. viviparus* larvae (Bovilis^®^ Huskvac, Intervet UK Ltd.) has been used successfully to control this parasite in the UK for many years (26). However, uptake of vaccination has declined with the increasing use of long-acting macrocyclic lactones for simultaneous control of lungworm and gastrointestinal nematodes. Suppressive drug treatment is not sustainable due to the strong selection for anthelmintic resistant individuals, with macrocyclic lactone resistant *D. viviparus* recently detected on a dairy farm in Scotland (23). The use of targeted anthelmintic treatment could improve the sustainability of parasite control, however lungworm outbreaks are severe and difficult to control, so strategic treatment approaches require sensitive and rapid diagnostic tests. A rapid detection tool using LAMP with a quantitative analysis (22) could be used to evaluate parasite burden and inform rational control, for example early treatment of at risk calves or post-treatment testing for drug efficacy. In addition to helping famers control lungworm in their herds, improving the accuracy of *D. viviparus* diagnosis in the field would inform epidemiological studies to better understand, predict, and control this highly pathogenic and economically important disease.

As a first step in developing a LAMP assay we investigated sequence variation at the conserved ITS2 locus in *D. viviparus* larvae from a farm in Scotland. Four sequence variations of the *D. viviparus* ITS2 DNA region (453–456 bp) were found in this study, from pooled PCR products amplified from five L_1_, all of which were detected by DviLAMP. Published ITS2 sequences from geographically separated *D. viviparus* (GenBank database, NCBI) showed additional genetic diversity, but the lack of full length 5.8S and ITS2 DNA sequence from these populations prevented assessment of primer hybridization in this study. Future work to examine a wider range of *D. viviparus* populations from different farms should be implemented to assess the sensitivity of the test in a wider context. In this study we found that the limit of detection was 0.5 ng with a 45 min reaction time. It may be possible to lower the limit of detection by increasing the reaction time to 90 min. In the real-time LAMP, we found that 1 pg of template could be detected after an 85–90 min reaction time. However, although increasing the reaction time can also increase the sensitivity of the reaction, this increase in sensitivity may not be practical for a rapid on-site test. Therefore, a balance between a user-friendly reaction time and detectable sensitivity, should be considered. A single L_1_ could be expected to have much less than 1 ng of DNA, however an active infection would likely have DNA from adults, multiple L_1_ and also recently ingested L_3_ from pasture. Therefore, a sensitivity of 0.5 ng or 1 pg is theoretically suitable for a field based diagnostic test.

We tested the ability of DviLAMP to differentiate between *D. viviparus* and other nematodes commonly found in bovine feces. The DviLAMP primers can amplify *D. viviparus*, *O. ostertagi* and *C. oncophora* ITS2 plasmids as observed by both gel electrophoresis and real-time amplification plots. However, the reactions with *O. ostertagi* and *C. oncophora* template produced a lower quantity of LAMP products when compared to the bright LAMP ladder band above 300 bp visible on gel electrophoresis for *D. viviparus*. The result indicates that a high quantity of longer LAMP products are produced only from *D. viviparus* template. However, the quantity of LAMP product alone is not sufficient evidence for differentiation between species, with amplification for all three species shown by real-time analyses. HRM analysis can be used to discriminate rumen fluke species (27), but although there is a peak of fluorescence signal at ~80°C for *D. viviparus*, further work is required to clearly differentiate non-target species in our study.

The amplification results for *O. ostertagi* and *C. oncophora* are consistent with the DNA alignment (Supplementary Figure S1) which showed the potential hybridized regions for the DviLAMP primers in forward sites, with a 100% match for F3 and F2, one mismatch in LF, and seven bases matching at the 3′ site of F1C. This reflects the high conservation of the DNA sequence in the 5.8S rDNA region. However, the sequence divergence in the remaining primers sited in the ITS2 region are expected to provide species specificity: there are no hits on either *O. ostertagi* or *C. oncophora* ITS2 sequences for the backward sites of DviLAMP primer sequences, B1C and LB, or for the 3′ nine bases of the B3 primer. We hypothesize the amplified LAMP product from non-target species showed a lesser effect on the colorimetry result (Figure 6C), due to an uncertain amount of H^+^ (LAMP by-product) produced by the less efficient reaction with incomplete loop structure formation from the primer hybridization on the forward site only. Consistent with this expectation, we show that the biotin-FITC tagging position on the primer or DNA probe strongly impacts the results, and correct positioning is essential for accurate diagnosis. The biotin tagged primer in scheme II specifically targets the *D. viviparus* ITS2 region only, so this is the key to the specificity of DviLAMP-LFD. This assumption is based on *in-silico* DNA sequence investigation. This could be explored further by the previously demonstrated LEC-LAMP assay design (28) to add further species specific discrimination of the LAMP products. However, the inclusion of this technology lay outside the current study’s scope, the focus of which was developing a species-specific assay for detecting bovine lungworm. This aim has been successfully demonstrated using LAMP-LFD. Nonetheless, it will be of great interest to explore this in future work. The detection of both *C. oncophora* and *O. ostertagia* ITS2 sequence by the lungworm primers developed herein opens the possibility to develop a triplex LEC-LAMP assay, using a similar methodology to that described previously for levamisole resistance (17) and bacterial species differentiation and antimicrobial resistance gene variants (28, 29). The LEC-LAMP assay design offers added advantage that it is also amenable to LFD end-point detection, although further work is necessary to optimize this (17).

For a user-friendly diagnostic test, the results should be obtained in the shortest time possible. Three different incubation times from 45 min to 90 min were tested, and the result indicated improving sensitivity for LAMP, colorimetry, and LFD by longer incubation. Incubation times for other published LAMP assays vary from about 15–60 min (15, 16) and the differing incubation times could be due to several factors in addition to DNA concentration, for example, DNA integrity and the presence of inhibitors from different extracted specimen types. This highlights that sample preparation/extraction before DNA amplification needs to be considered. To develop DviLAMP as a point-of-care diagnostic test additional steps relating to sample preparation need to be undertaken. The assay could be used to detect larvae in feces, which would require a simple method to isolate *D. viviparus* DNA and remove potential inhibitors. DviLAMP could potentially be developed to detect eggs and/or larvae directly in bovine nasal mucus, which would potentially allow earlier detection of infection than using feces. However, the amount of free *D. viviparus* DNA in mucus of infected calves is currently unknown so this may also require a DNA extraction step. Farmer preference and practicalities of handling youngstock will also dictate the most appropriate sampling approach. In addition, LAMP could be further developed to detect anthelmintic resistance mutations, as has been shown for levamisole resistant *Haemonchus contortus* (Rudolphi, 1803) Cobb, 1898 using colorimetric, SNP specific enzymatic cleavage, and restriction analysis (17, 28). A future application of LAMP in point-of-care and field-based detection could combine a microfluidic chip (for mixing reagents and incubation) with a convenient measurement (e.g., colorimetry, lateral flow, electrochemistry) and digital data processing, to present, analyze, and store results (30–33). In addition, there is growing interest in the potential for combining LAMP with portable sequencing technologies such as Oxford Nanopore (34). Thus, our newly designed and validated LAMP primer set for the detection of *D. viviparus* ITS2 could be integrated into a future farm-side sequencing assay.

In conclusion, we have developed a novel proof of concept LAMP assay for *D. viviparus* with various methods of end point detection, including colorimetry, gel electrophoresis, real-time with HRM, and LFD. Its application with colorimetry and LFD can detect target DNA specifically and sensitively down to a concentration of 0.5 ng within 45–60 min of incubation at 64°C. Although non-target nematodes, *O. ostertagi* and *C. oncophora*, can be detected by gel electrophoresis and real-time, they were effectively discriminated from the positive *D. viviparus* result in colorimetry and LFD assays. Therefore, the DviLAMP represents a major step in the development of a field-based diagnostic tool for *D. viviparus* to improve parasite control and livestock health.

## Data Availability

The data presented in the study are deposited in the Nucleotide database (https://www.ncbi.nlm.nih.gov/nuccore/), accession numbers: PP970511-PP970515, PP968975-PP968977.

## Footnotes

^1^ https://primerexplorer.jp/lampv5e/index.html

## Supplementary material

SUPPLEMENTARY FIGURE S1 ITS2 with flanking 5.8S and 28S rDNA sequences alignment of *Ostertagia ostertagi* and *Cooperia oncophora* highlighted with different primers DNA sequences for the DviLAMP; variable sites in the DNA sequence are presented as red letters; gray letters indicating non-hybridized primer region.

SUPPLEMENTARY FIGURE S2 Detection of various DviITS2 plasmid variations (clone 5, 7, 9, and 14) at 10 ng using the DviLAMP (60 min reaction time) with lateral flow dipstick (LFD) **(A)**, colorimetry **(B)**, and gel electrophoresis **(C)**. L, DNA ladder; and N, negative control.
