# Supplementary material for: Development of a loop-mediated isothermal amplification detection assay for Dictyocaulus viviparus (Bloch, 1782) lungworm: DviLAMP
Source: Front Vet Sci. 2024 Oct 4;11:1454065. doi: 10.3389/fvets.2024.1454065 (PMC11486680; doi:10.3389/fvets.2024.1454065)
Supplement: Supplementary file 3 [file Table_1.DOCX]

**Supplementary material**

**Supplementary Tables**

**Supplementary Table S1** BLASTn result and information for DNA sequences in this study. The % identity refers to nucleotide matches with the identified DNA sequences in the NCBI database. bp =base pair.

| BLASTn result | | | | | | | |
| --- | --- | --- | --- | --- | --- | --- | --- |
| Name | Length (bp) | Identified species | | % Identity | | | |
|  |  |  |  | Lowest | | Highest | |
| Dvi_ITS2_05 | 596 | *Dictyocaulus viviparus* | | 98.02 | | 99.69 | |
| Dvi_ITS2_06 | 596 | *Dictyocaulus viviparus* | | 98.02 | | 99.69 | |
| Dvi_ITS2_07 | 593 | *Dictyocaulus viviparus* | | 97.22 | | 100 | |
| Dvi_ITS2_09 | 596 | *Dictyocaulus viviparus* | | 98.21 | | 99.78 | |
| Dvi_ITS2_14 | 596 | *Dictyocaulus viviparus* | | 97.62 | | 100 | |
| Oos_ITS2_13 | 378 | *Ostertagia ostertagi* | | 98.14 | | 100 | |
| Con_ITS2_16 | 381 | *Cooperia oncophora* | | 99.21 | | 99.74 | |
| DNA regions on the sequence | | | | | | | |
| Name | 5.8S rDNA  position | Length  (bp) | ITS2  position | Length  (bp) | 28S rDNA  position | | Length  (bp) |
| Dvi_ITS2_05 | 1 ... 87 | 87 | 88 … 543 | 456 | 544 … 596 | | 53 |
| Dvi_ITS2_06 | 1 ... 87 | 87 | 88 … 543 | 456 | 544 … 596 | | 53 |
| Dvi_ITS2_07 | 1 ... 87 | 87 | 88 … 540 | 453 | 541 … 593 | | 53 |
| Dvi_ITS2_09 | 1 ... 87 | 87 | 88 … 543 | 456 | 544 … 596 | | 53 |
| Dvi_ITS2_14 | 1 ... 87 | 87 | 88 … 543 | 456 | 544 … 596 | | 53 |
| Oos_ITS2_13 | 1 ... 87 | 87 | 88 … 325 | 238 | 326 … 378 | | 53 |
| Con_ITS2_16 | 1 ... 87 | 87 | 88 … 328 | 341 | 329 … 381 | | 53 |
